# Hepatocellular Carcinoma Drug-Eluting Bead Transarterial Chemoembolization (DEB-TACE): Outcome Analysis Using a Model Based On Pre-Treatment CT Texture Features

**DOI:** 10.3390/diagnostics11060956

**Published:** 2021-05-26

**Authors:** Marcello Andrea Tipaldi, Edoardo Ronconi, Elena Lucertini, Miltiadis Krokidis, Marta Zerunian, Tiziano Polidori, Paola Begini, Massimo Marignani, Federica Mazzuca, Damiano Caruso, Michele Rossi, Andrea Laghi

**Affiliations:** 1 Department of Surgical and Medical Sciences and Translational Medicine, Sapienza-University of Rome, 00189 Rome, Italy; marta.zerunian@gmail.com (M.Z.); michele.rossi@uniroma1.it (M.R.); andrea.laghi@uniroma1.it (A.L.); 2 Department of Radiology, Sant’Andrea University of Hospital La Sapienza, 00189 Rome, Italy; edoardo.roncon1@gmail.com (E.R.); Elena.lucertini@gmail.com (E.L.); tiziano.polidori@yahoo.it (T.P.); 3 Department of Radiology, Areteion Hospital, National and Kapodistrian University of Athens, 11528 Athens, Greece; mkrokidis@hotmail.com; 4 Department of Diagnostic, Interventional and Pediatric Radiology, Inselspital Bern University Hospital, University of Bern, 3010 Bern, Switzerland; 5 Department of Liver Diseases Section, AOU Sant’Andrea Hospital, University of Hospital La Sapienza, 00189 Rome, Italy; paolabegini@gmail.com (P.B.); mmarignani@hotmail.com (M.M.); 6 Department of Clinical and Molecular Oncology-Sapienza, University of Rome, Sant’Andrea University Hospital, via di Grottarossa 1035, 00189 Rome, Italy; federica.mazzuca@uniroma1.it; 7 Department of Radiological Sciences, Oncological and Pathological Sciences, University of Rome Sapienza, Sant’Andrea University Hospital, 00189 Rome, Italy; dcaruso85@gmail.com

**Keywords:** hepatocellular carcinoma, radiomics, texture analysis, TACE, interventional oncology

## Abstract

(1) Introduction and Aim: The aim of this study is to investigate the prognostic value, in terms of response and survival, of CT-based radiomics features for patients with HCC undergoing drug-eluting beads transarterial chemoembolization (DEB-TACE). (2) Materials and Methods: Pre-treatment CT examinations of 50 patients with HCC, treated with DEB-TACE were manually segmented to obtain the tumor volumetric region of interest, extracting radiomics features with TexRAD. Response to therapy evaluation was performed basing on post-procedural CT examination compared to pre-procedural CT, using modified RECIST criteria for HCC. The prognostic value of texture analysis was evaluated, investigating the correlation between radiomics features, response to therapy and overall survival. Three models based on texture and clinical variables and a combination of them were finally built; (3) Results: Entropy, skewness, MPP and kurtosis showed a significant correlation with complete response (CR) to TACE (all *p* < 0.001). A predictive model to identify patients with a high and low probability of CR was evaluated with an ROC curve, with an AUC of 0.733 (*p* < 0.001). The three models built for survival prediction yielded an HR of 2.19 (95% CI: 2.03–2.35) using texture features, of 1.7 (95% CI: 1.54–1.9) using clinical data and of 4.61 (95% CI: 4.24–5.01) combining both radiomics and clinical data (all *p* < 0.0001). (4) Conclusion: Texture analysis based on pre-treatment CT examination is associated with response to therapy and survival in patients with HCC undergoing DEB-TACE, especially if combined with clinical data.

## 1. Introduction

Hepatocellular carcinoma (HCC) is mainly associated with chronic liver disease and it is the fifth most common malignant tumor worldwide and the second leading cause of cancer-related mortality [1]. Transarterial chemoembolization (TACE) is the first line treatment for HCC at intermediate stage of disease, according to the Barcelona Clinic Liver Cancer (BCLC) staging system, but it was recently demonstrated to be an effective procedure at any HCC stage [2]. Nowadays, according to the European Association for the Study of the Liver (EASL) guidelines, TACE is also widely accepted as a neoadjuvant therapy before liver transplantation to downstage the tumor burden [3].

There are two different TACE techniques. Conventional TACE (cTACE) is the most common modality performed worldwide and it employs lipiodol suspension and gelatine sponge particles. It is also possible to perform TACE with drug-eluting beads (DEB-TACE). Currently, there is no scientific evidence to demonstrate the superiority of one technique over the other in terms of tumor response, survival, safety and cost-effectiveness [2,4]; nevertheless, TACE outcomes are mainly assessed with imaging such as computed tomography (CT) and magnetic resonance imaging (MRI) and this is the aspect that most studies are focused on [5].

Radiomics is an advanced analysis method which can be applied on CT, MRI and other imaging techniques, allowing to extrapolate quantitative features from regions or volumes of interest (ROI/VOI) in diagnostic images [6]. Application of radiomics in oncology for tumor characterization, response assessment, prediction of response to therapies and survival prediction has been widely investigated in the literature with good results, including in HCC [7].

As far as the prediction of response to treatments in HCC is concerned, radiomics has been applied on CT and MRI pre-treatment images in some studies, identifying specific radiomic features that were significantly correlated with response to surgery [8], radiofrequency ablation [9], chemotherapy with Sorafenib [10] and TACE combined with Sorafenib sistemic therapy [11]. Some radiomic features were also demonstrated to be valuable predictive factors of response to TACE as a single HCC treatment and most of them were based on MRI [12,13,14,15]. Furthermore, Kim et al. [16] identified a combined score model integrating clinical data and texture analysis, with a good predictive value on survival after cTACE.

The purpose of this study was to assess long term outcomes of DEB-TACE in a single center population and to investigate whether pretreatment CT-based radiomics features are associated with response and survival.

## 2. Materials and Methods

### 2.1. Patient Population

Patients that underwent treatment with DEB-TACE for radiologically confirmed (LI-RADS 5) [17] HCC in our center between 2009 and 2014 were reviewed. We included only patients treated with DEB-TACE instead of cTACE because they were much more numerous in our series, allowing a more homogeneous study. Since its introduction, we preferred DEB-TACE over C-TACE due to its lower sistemic effects. Inclusion criteria were: (a) Treatment of HCC with DEB-TACE as a first line of treatment; (b) treatment with DEB-TACE as a second line treatment only when previous treatment was performed at least 12 months earlier and concerned different lesions that were treated only with percutaneous thermal ablation; (c) pre and triphasic contrast (arterial, portal, delayed) CT scan performed at least 60 days before treatment; and (d) pre and triphasic contrast CT scan performed within 3 months after treatment. Exclusion criteria were: (a) non-suitable target lesions for segmentation; (b) presence of portal thrombosis or extra-hepatic disease; (c) lack of availability of clinical information or survival data; and (d) not HCC-related death causes. Applying these criteria, out of the initially 96 recruited patients, 50 were finally selected. Demographic and clinical data of the included population are shown in Table 1.

### 2.2. CT Acquisition and Evaluation

CT examinations before and after treatment were performed by using a 256-slice CT (Brilliance iCT 256, Philips Healthcare, Eindhoven, Netherlands), with a tube voltage of 120 kVp.

All CT scans were performed in a cranio-caudal direction, with the patient in a supine position. Dynamic scans were performed using a bolus-tracking software program, with the placement of a 150 HU-threshold region-of-interest (ROI) within the abdominal aorta at the level of the celiac tripod. All CT examinations included a pre-enhanced phase, a parenchimal arterial phase acquired 15 s after reaching the threshold of the ROI, a portal venous phase 70 s after the threshold and a later phase after 180 s.

Response to therapy evaluation was performed by two radiologists in consensus, with 5 and 12 years of experience in the field of interventional radiology, and was based on a post-procedural CT examination performed within 3 months from the treatment and compared to pre-procedural CT, using modified RECIST (mRECIST) criteria for hepatocellular carcinoma [18], identifying as possible results progressive disease (PD), stable disease (SD), partial response (PR) or complete response (CR).

### 2.3. Texture Analysis

**Figure 1:**
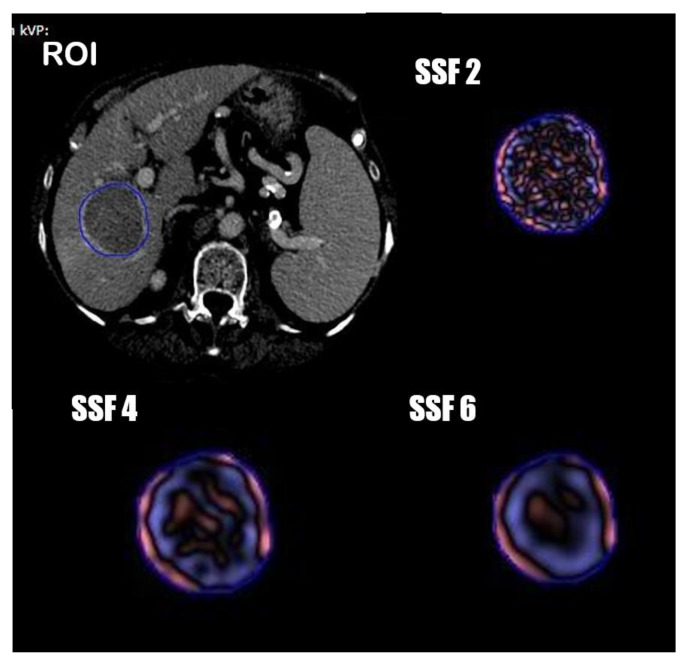
Segmentation process and spatial scale filters (SSF) application.

One radiologist and one resident in consensus manually segmented the tumor volumetric region of interest (VOI) on axial portal phases of the pre-treatment CT images, always considering the arterial phase to compare the geometrical shape of the lesion. (Figure 1) Radiomics features were extracted from the obtained VOIs and analyzed with TexRAD, a proprietary software algorithm (TexRAD Ltd, Somerset, England, United Kingdom).The spatial scale filter (SSF) value was altered between 0 and 6, extrapolating CT intensity features of three different sizes: fine (between 0 and 2 mm), medium (between 3 and 4 mm) and coarse (between 5 and 6 mm). Then, the following histogram parameters were extracted with every filter: mean, standard deviation, mean value of positive pixels (MPP), skewness, entropy, and kurtosis. In the final analysis, 18 features were extracted and analyzed.

### 2.4. Treatment Modality

The treatment was performed with selective catheterization of the tumoral feeding arteries and injection of DC Bead™ microspheres 100–500 µm (Boston Scientific) associated to 50 to 150 mg of doxorubicin, depending on the tumor size and vascularization, until a complete blockage of the flow of tumor feeding branches was achieved. Procedures were performed by three interventional radiologists with more than 5 years of experience. The angiographic suit was equipped with a flat panel image intensifier, Digital Innova 2000 (GE, New York, NY, USA). According to the operator preference, a 3DCT was performed simultaneously in order to improve the identification of target tumor vessels.

### 2.5. Statistical Analysis

A normality test was performed on all continuous variables. Continuous variables were expressed as medians and standard deviation, while categorical variables were expressed as counts and percentages.

Univariate analysis was performed for radiomics and clinical features using an independent T-test for variables with a normal distribution, and a Mann–Whitney U test for non-normally distributed variables.

A Pearson chi-squared test was used for categorial variables.

Continuous variables were dichotomized around an optimal cut-off via ROC using the Youden index.

A logistic regression with a forward stepwise selection and a bootstrap internal validation was used to construct the model to predict treatment response, and model performance was evaluated using ROC curves. Survival models were constructed using multivariate Cox regression analysis and Kaplan–Meier survival analysis. A *p* value of 0.05 was considered statistically significant.

The software BM SPSS Statistics for Windows, Version 24.0 (IBM Corp., Armonk, NY, USA) was used for the statistical analysis.

## 3. Results

### 3.1. Patient Characteristics

The mean patient age of the 50 patients was 70 ± 9 years, and the majority were male (*n* = 42; 84%). Average weight was 69.9 ± 10.4 Kg. Most patients were BCLC stage A (*n* = 26) by the time of the procedure, the rest were BCLC stage B (*n* = 22) or C (*n* = 2). Child-Pugh class liver function was mostly A (*n* = 36.72%), the rest was class B. Ten patients presented ascites at the time of the procedure. Mean albumin level was 3.6 ± 0.4 g/dL, mean bilirubin value was 1.3 ± 0.5 mg/dL, mean INR 1.21 ± 0.1 and mean α-fetoprotein level was 345 ± 751 ng/mL. A total of 30 patients (60%) had a single lesion, whilst 10 patients had 2 lesions and 10 patients had more than 2 lesions, with a maximum of 10 lesions in a single patient. The mean lesion size was 42 ± 20 mm. Sixteen patients were previously treated with thermal ablation and 10 patients had lesion within the Milan criteria but were not considered fit for surgery.

Population characteristics are shown in Table 1.

### 3.2. Treatment Effectiveness and Overall Survival

At post-TACE contrast enhanced CT, 22 (44%) patients showed a CR, while 20 (40%) had a PR, 6 (12%) showed a SD and 2 (4%) suffered from a PD. Mean OS was 934 days, and 8 patients were censored when conducting the analysis due to being alive at the endpoint of 1825 days (5 years). Treatment response revealed a significant correlation with OS (Log Rank Test, *p* = 0.002) with a mean OS of 1032 days for CR, 694 days for PR, 129 days for SD and 227 days for PD (Table 2).

### 3.3. Variables and Association with Response to TACE

**Figure 2:**
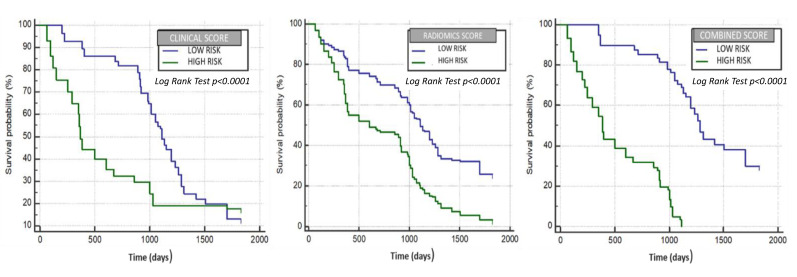
Clinical variables, radiomic features and combined clinical and radiomic features model performance in evaluating OS.

After univariate analysis, texture analysis features showing a significant correlation with CR were standard deviation (*p* = 0.002), entropy, skewness, MPP and kurtosis (*p* < 0.001). ROC curves were performed to identify cut-offs for these variables (Table 3), and after dichotomization, a multivariate logistic regression analysis with stepwise forward selection and Bootstrap internal validation was performed. Optimal cut-offs of significant variables in the model (entropy, skewness, MPP and kurtosis) with OR identified in the multivariate analysis were used to construct a score to identify two classes of patients with a high and low probability of obtaining a CR after TACE (Table 4). Performance of the model was evaluated using an ROC curve (AUC 0.733, *p* < 0.001, Figure 2).

### 3.4. Development of the Radiomic, Clinical Survival and Combined Model

We considered the survival time as the response variable and performed an univariable analysis, identifying significant radiomics and clinical variables. Next, continuous variables were dichotomized via ROC curves, identifying optimal cut-off values using the Youden index, and are showed in Table 5. A Multivariate Cox regression analysis was performed using texture and clinical features alone and in combination, as shown in Table 6, Table 7 and Table 8. Significant variables from the multivariate analysis were used to build three different scores by the linear combination with beta coefficients resulting from the multivariate Cox regression models. When looking at the model developed using both clinical variables and radiomic features, an age older than 55 years, female sex, albumin serum levels ≤3.4 g/dL, birilubin serum levels ≤1.7 mg/dL, AFP levels <400 ng/mL, a single lesion, a maximum axial diameter of the target lesion ≤46 mm and the BCLC and Child–Pugh stage were used from the former, and a mean >10.59, entropy ≤4.8, skewness ≤0.02 and MPP >45.82 from the latter. Patients were then stratified into two classes using the median value of each score model, and correlation of models with overall survival was assessed using Kaplan–Meier analysis. Survival differences between the groups were calculated as hazard ratios (HRs) for each model by use of log-rank tests. In the radiomics score model (Figure 2, the median survival of the high-risk group was 602 days, and that of the low-risk group was 1111 days, yielding an HR of 2.19 (95% CI: 2.03–2.35) (*p* < 0.0001). In the clinical model (Figure 2), the median survival of the high-risk group was 370 days, and that of the low-risk group was 1111 days, yielding an HR of 1,7 (95% CI: 1.54–1.9) (*p* < 0.0001). In the combined score model (Figure 2), the median survival of the high-risk group was 387 days, whereas that of the low-risk group was 1285 days, with an HR of 4.61 (95% CI: 4.24–5.01) (*p* < 0.0001).

## 4. Discussion

This study assessed the feasibility of radiomics in the prediction of response and survival to DEB-TACE for patients with HCC.

Data of texture analysis extracted from TextRad software (entropy, skewness, MPP and kurtosis) were able to identify two groups of patients with a different probability of response to treatment, evaluated with the mRECIST criteria, respectively with a low and a high probability of obtaining a complete response, corresponding with a better outcome and survival. Moreover, the model built to evaluate the radiomics data association with survival was able to identify two groups of patients with different median survival times (1111 vs. 602 days) as shown in the Kaplan–Meier graphic: an HR of 2.19 (95% CI: 2.03–2.35) (*p* < 0.0001) while the model built with the only clinical data yield an HR of 1.7 (95% CI: 1.54–1.9) (*p* < 0.0001) and a median OS of 1111 vs. 370 days. In the end, we combined both the radiomics and clinical models to construct a combined model that provided an even better estimation of survival time (median OS of 1285 vs. 387 days) with an HR of 4.61 (95% CI: 4.24–5.01) (*p* < 0.0001).

The most significant texture parameters associated with treatment outcome were entropy, skewness and kurtosis. In particular, kurtosis was statistically lower in patients with a better response; elevated values of kurtosis describe the pixel histogram to be more peaked or pointier than a Gaussian distribution, meaning that patients’ nodules with a better prognosis might have more regular nodular architecture reflected in a more homogeneous pixel distribution. On the other hand, ROI asymmetry is expressed by skewness and these values showed the higher heterogeneity in patients with a worse prognosis. A tumor a with high textural heterogeneity often has a poor prognosis and hence can negatively affect survival [19,20]. Even the entropy which is another manifestation of higher tumor heterogenicity showed lower values in patients with a better prognosis. This result is in line with the previously published paper by Cozzi et al. [21] in which the entropy was found to be an independent predictor of OS in patients with advanced HCC undergoing systemic therapy with sorafenib.

Regarding clinical variables, our study revealed that different variables, such as Child–Pugh score, α-fetoprotein level, HCC size and number were significant in the clinical score model, according with existing studies [22,23].

As was recently reported, those models with a better performance are those in which radiomic features are computed with clinical data [16,24,25]. Our combined model confirmed the radiomic pretreatment CT features together with clinical variables, and could be a good prognostic biomarker of the overall survival of patients suffering of HCC treated with DEB-TACE.

Texture analysis has been introduced in medical oncologic imaging as a noninvasive imaging biomarker with the aim to extract quantitative parameters from images to provide an objective characterization of the lesions [26]. Several studies have already tested this approach on different neoplasms and in particular on lung cancer, showing some interesting results in the discrimination of malignancy in pulmonary lesions [26,27,28]. Moreover, texture analysis is being demonstrated to be a potential biomarker for the prediction of response to treatments and therapy [21]. In particular regarding HCC, Kim et al. first reported the prognostic value of a model based on pre-treatment CT-Texture analysis with better performance when combining clinical and radiomic variables. The authors reported excellent results of the model based on a second-order texture analysis and in evaluating survival after cTACE. In our study, however, pre-treatment CT-texture analysis was evaluated using a first-order statistical analysis with the well-known Texrad software and to evaluate patients treated with DEB-TACE in terms of survival but also in terms of response to treatment. In our view, lipiodol embolization could lead to post treatment CT interpretation pitfalls due to the high attenuation of the lipiodol itself. This pitfall may be overcome with the use of drug-eluting beads that do not influence the postoperative scans in terms of CT density. Moreover, our study demonstrated that texture analysis is significantly associated with response to DEB-TACE evaluated with the mRECIST criteria (model ROC curve (AUC 0.733, *p* < 0.001).

This study has some inevitable limitations. Firstly, the population sample is limited; however, it concerns a very specific treatment, and comes from a single center only. Secondly, due to in consensus reading, there is lack of inter-reader agreement analysis. Thirdly, the use of a first-order statistical analysis allowed a simpler prediction model, and the identified cut-off values for the relevant parameters might be different in a different clinical scenario due to the lack of external validation or an internal validation cohort.

Nevertheless, our results are encouraging and confirm the prognostic value of Texture analysis in the pretreatment evaluation of response and survival to DEB-TACE for HCC, especially when combined with clinical data. This information may be useful to gain a more precise personalized oncologic approach that is mainly based on outcome predictions and personalized treatment than “one-fits-all” blanket treatments.

Further studies with a prospective design are required to further investigate the role of texture analysis in this scenario. Moreover, this is also a call for all radiologists and interventional radiologists towards a standardization of pre- and post-treatment imaging and data collection and towards high quality data registries [29]. Machine learning is already an everyday tool and we need to implement it for the benefit of our patients.

## Figures and Tables

**Table 1:** Baseline Patient Characteristics.

Characteristic	Value	
Age	Years, mean *± SD*	70 ± 9
Sex	*m*	42 (84%)
	*f*	8 (6%)
Albumin	g/dL, mean *± SD*	3.6 ± 0.4
Bilirubin	mg/dL, mean *± SD*	1.3 ± 0.5
AFP	ng/mL, mean *± SD*	345 ± 751
BCLC stage	A	26 (52%)
	B	22 (44%)
	C	2 (4%)
Child-Pugh	A	36 (72%)
	B	14 (28%)
Maximum Diameter	mm, mean *± SD*	42 ± 21
n° of lesions	1	30 (60%)
	2	10 (20%)
	3+	10 (20%)

**Table 2:** TACE Treatment Response.

Category	*n*° and Percentage	OS (Mean, Days)
CR	22 (44%)	1032
PR	20 (40%)	694
SD	6 (12%)	129
PD	2 (4%)	227

CR—complete response; PR–partial response; SD—stable disease; PD–progressive disease.

**Table 3:** ROC Curves for Radiomics Features. Association with CR.

Radiomic Feature	AUC	Cut-Off	*p* Value
Skewness	0.713	≤0.14	<0.001
Entropy	0.621	≤4.78	<0.001
Kurtosis	0.614	≤−0.059	<0.001
MPP	0.561	≤48.189	<0.001
SD	0.544	≤35.659	0.002

MPP—mean of positive pixel; SD—standard deviation.

**Table 4:** Logistic Regression with Bootstrap Validation for Radiomics Features Association with Treatment Response.

			95% C.I. EXP(B)		
	*p* Value	Exp(B)	Lower	Upper	
Skewness	0.001	3.936	3.120	4.966	
Entropy	0.001	1.992	1.455	2.727	
Kurtosis	0.001	1.692	1.367	2.094	
MPP	0.001	1.958	1.574	2.436	
SD	0.998	0.999	0.726	1.376	
Model Performance			Hosmer–Lemeshow Goodness-of-fit		
Likelihood Log −2	R-Square Cox and Snell	R-Square Nagelkerke	Chi-square	df	*p* value
2069.452	0.147	0.204	13.211	8	0.105

**Table 5:** Univariate analysis of radiomics and clinical variables for OS.

Variable	AUC	Cut-Off	*p* Value
Mean	0.609	>10.59	<0.001
Entropy	0.538	≤4.8	<0.001
Skewness	0.547	≤0.02	<0.001
MPP	0.552	>45.82	<0.001
Age. per year	0.545	>55 years	<0.001
Sex-female			<0.001
Albumin. g/dL	0.707	≤3.4 g/dL	<0.001
Birilubin. mg/dL	0.559	≤1.7 mg/dL	<0.001
AFP. ng/mL	<400 ng/mL		<0.001
Single lesion	0.654		<0.001
Maximum Diameter. mm 0.589		≤46 mm	<0.001
BCLC stage A			<0.001

**Table 6:** Cox regression analysis—radiomic feature association with OS.

	Univariate	Multivariate		95% C.I. EXP(B)	
	*p* Value	*p* Value	Exp(B)	Lower	Upper
Mean	<0.001	<0.001	1.646	1.510	1.795
Entropy	<0.001	<0.001	0.778	0.721	0.841
Skewness	<0.001	<0.001	0.799	0.744	0.859
MPP	<0.001	<0.001	0.737	0.676	0.803

MPP—mean of positive pixel.

**Table 7:** Cox regression analysis—clinical variable association with OS.

	Univariate	Multivariate		95% C.I. EXP(B)	
	*p* Value	*p* Value	Exp(B)	Lower	Upper
Age	<0.001	< 0.001	1.766	1.433	2.177
Sex	<0.001	<0.001	0.019	0.014	0.026
Albumin	<0.001	<0.001	0.058	0.050	0.067
Bilirubin	<0.001	<0.001	1.359	1.165	1.585
AFP	<0.001	<0.001	6.671	5.817	7.650
*n*°	<0.001	<0.001	0.253	0.212	0.303
Maximum Diameter	<0.001	<0.001	0.327	0.270	0.397
BCLC A	<0.001	<0.001	0.131	0.095	0.180
BCLC B	<0.001	<0.001	0.282	0.188	0.423
Child–Pugh A	<0.001	<0.001	1.512	1.274	1.794

AFP—α Feto Protein; BCLC—Barcelona Clinic Liver Cancer. BCLC stage C was excluded from the model due to collinearity.

**Table 8:** Cox regression analysis—combined radiomic features and clinical variable association with OS.

	Univariate	Multivariate		95% C.I. EXP(B)	
	*p* Value	*p* Value	Exp(B)	Lower	Upper
Mean	<0.001	0.001	1.186	1.072	1.313
Entropy	<0.001	<0.001	1.351	1.241	1.471
Skewness	<0.001	<0.001	0.663	0.612	0.718
MPP	<0.001	0.006	0.875	0.796	0.963
Age	<0.001	<0.001	2.203	1.787	2.717
Sex	<0.001	<0.001	0.027	0.020	0.036
Albumin	<0.001	<0.001	0.052	0.044	0.060
Bilirubin	<0.001	<0.001	1.347	1.158	1.568
AFP	<0.001	<0.001	7.569	6.552	8.745
*n*°	<0.001	<0.001	0.227	0.191	0.272
Maximum Diameter	<0.001	<0.001	0.249	0.204	0.303
BCLC A	<0.001	<0.001	0.136	0.098	0.188
BCLC B	<0.001	<0.001	0.228	0.152	0.343
Child–Pugh A	<0.001	<0.001	1.733	1.458	2.059

MPP—mean of positive pixel; AFP—α Feto Protein. BCLC—Barcelona Clinic Liver Cancer BCLC stage C was excluded from the model due to collinearity.

## Data Availability

The data presented in this study are available on request from the corresponding author. The data are not publicly available due to privacy and Local Institution policy.
